# Predicting Clinical Outcome in Acute Ischemic Stroke Using Parallel Multi-Parametric Feature Embedded Siamese Network

**DOI:** 10.3390/diagnostics10110858

**Published:** 2020-10-22

**Authors:** Saira Osama, Kashif Zafar, Muhammad Usman Sadiq

**Affiliations:** Department of Computer Science, National University of Computing and Emerging Sciences, 852-B Milaad St, Block B Faisal Town, Lahore 54000, Pakistan; saira.osama@nu.edu.pk (S.O.); usman.sadiq@nu.edu.pk (M.U.S.)

**Keywords:** acute ischemic stroke, siamese network, machine learning, deep learning, imbalance, multi-parametric MRI, feature embedding

## Abstract

Stroke is the second leading cause of death and disability worldwide, with ischemic stroke as the most common type. The preferred diagnostic procedure at the acute stage is the acquisition of multi-parametric magnetic resonance imaging (MRI). This type of imaging not only detects and locates the stroke lesion, but also provides the blood flow dynamics that helps clinicians in assessing the risks and benefits of reperfusion therapies. However, evaluating the outcome of these risky therapies beforehand is a complicated task due to the variability of lesion location, size, shape, and cerebral hemodynamics involved. Though the fully automated model for predicting treatment outcomes using multi-parametric imaging would be highly valuable in clinical settings, MRI datasets acquired at the acute stage are mostly scarce and suffer high class imbalance. In this paper, parallel multi-parametric feature embedded siamese network (PMFE-SN) is proposed that can learn with few samples and can handle skewness in multi-parametric MRI data. Moreover, five suitable evaluation metrics that are insensitive to imbalance are defined for this problem. The results show that PMFE-SN not only outperforms other state-of-the-art techniques in all these metrics but also can predict the class with a small number of samples, as well as the class with high number of samples. An accuracy of 0.67 on leave one cross out testing has been achieved with only two samples (minority class) for training and accuracy of 0.61 with the highest number of samples (majority class). In comparison, state-of-the-art using hand crafted features has 0 accuracy for minority class and 0.33 accuracy for majority class.

## 1. Introduction

**Figure 1:**
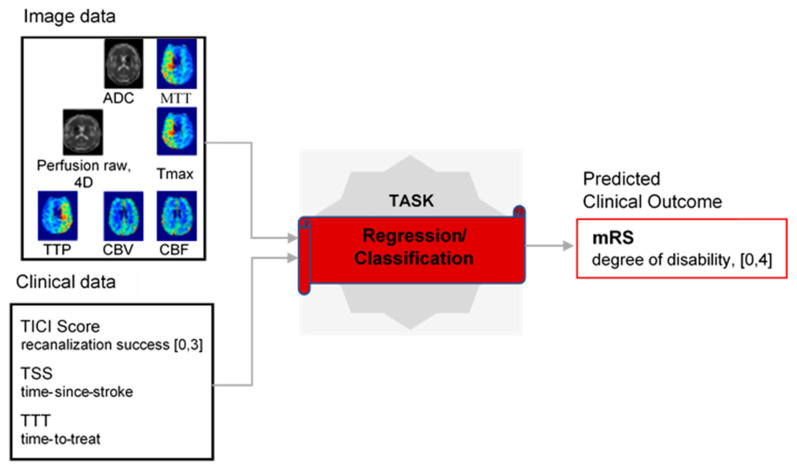
Problem of predicting clinical outcome.

Stroke is the second leading cause of death and a major cause of disability worldwide [1]. Annually, 15 million people worldwide suffer a stroke that results in permanent disability, placing a burden on family and community [2]. Most strokes (80%) occur by an unexpected blockage of arteries carrying blood to the brain, causing ischemia, and are called ischemic strokes. The ischemia is the lack of oxygen in tissues. As a result, tissues start to die quickly in the next few minutes. The dead tissue is called “core”. The salvageable tissue is termed as “penumbra” and it is the target for reperfusion therapies. The affected area of the brain, the stroke lesion, go through several disease stages that can be categorized as acute (0–24 h), sub-acute (24 h–2 weeks) and chronic (>2 weeks) according to the time passed since stroke onset [3]. The preferred diagnostic procedure at acute stage involves the acquisition of multi-parametric magnetic resonance imaging (MRI). The possible options for treatment are largely limited to reperfusion therapies known as thrombolysis and thrombectomy, which must be managed not later than four to six hours after the symptom onset [4]. This treatment is associated with high risk of intracranial hemorrhage. The success of the intervention is assessed via the standardized thrombolysis in cerebral infarction (TICI) grading system [5]. As this intervention is risky, clinicians are interested in measuring the treatment outcome of a patient, i.e., to see the possible benefits of treatment vs. risk. The gold standard when measuring the outcome for a patient is either in the form of lesion outcome or clinical outcome. Lesion outcome is determined by a three-month follow-up MR scan showing the lesion. Clinical outcome after three months is in the form of modified Rankin Scale (mRS). The mRS runs from 0–6 showing the degree of disability (0–5) and death (6) [6]. The problem of predicting the clinical outcome of treated patients is shown in Figure 1.

Clinical outcome prediction in treated patients is complex because it involves various clinical and imaging biomarkers. The challenge is to integrate these biomarkers into an outcome prediction model as the relationship amongst a patient’s stroke presentation, clinical parameters, treatment scores and functional outcomes is not well defined yet. Recent machine learning techniques, such as deep learning, have been used in the field of cerebrovascular disorders [7] and have the potential to solve the important problem of outcome prediction in acute ischemic stroke [8]. Machine learning models have been created to predict the outcome after reperfusion therapy using neuroimaging [9]; however, most of these studies focused on the prediction of lesion outcome as compared to clinical outcome [10]. We focused only on clinical outcome prediction using multi-parametric MRI imaging data acquired at the acute stage before any treatment decision, as it is the preferred imaging for diagnosis and treatment. These imaging data are fed into parallel multi-parametric feature embedded siamese network (PMFE-SN), which is a novel deep learning end-to-end model presented in this paper. Deep learning techniques require large amount of data, so there is a need of robust and large datasets in this area [9,10]. In this regard, Maier et al. [11,12] reported the urgent need of comparability of outcome prediction models due to the variability in the nature of data, outcomes and metrics used for comparison in various studies. For that matter, they launched benchmark datasets named ISLES 2016 and ISLES 2017. ISLES 2017 dataset is an extension of ISLES 2016 dataset. The challenge results on ISLES 2016 dataset for clinical outcome prediction are sub-optimal due to very small number of samples [12]. This research presents PMFE-SN, an automated end-to-end method for predicting treatment outcome even with less and highly skewed MRI data. The developed model helps in reducing bias towards majority class, as well as learning from very few samples, i.e., only two samples for training. This is the first attempt to solve this problem using ISLES 2017 dataset.

The major contributions of this research include combining multi-parametric 3D MRI images in an end-to-end deep learning architecture model, development of multi-parametric feature embedding in siamese network for handling scarcity of multi-parametric MRI data, introducing two-stage balancing strategy for solving class imbalance problems and defining evaluation metrics insensitive to imbalance. Metrics used in previous approaches could be misleading in the assessment of model performance and in turn could misguide clinicians in making treatment decisions.

The following section—Section 2—presents the literature review highlighting the drawbacks of the research work done so far in this area. Section 3 provides the details of the developed methodology. Experimental setup is explained in Section 4. The last section—Section 5—discusses various experiments and results in comparison to other state-of-the-art methods.

## 2. Literature Review

Due to the difficult nature of the problem, most papers have dichotomized the clinical outcome score in different forms. Dichotomizing refers to the process of converting mRS (0–6) to a two-class problem. This section is divided into dichotomized output-based papers and non-dichotomized output-based papers.

### 2.1. Dichotomized Output

Ho et al. [13] built the support vector machine (SVM) using demographic and clinical data from University of California, Los Angeles (UCLA) Research Electronic Data Capture (Redcap) with 190 patients for predicting discharge mortality. Synthetic Minority Over-sampling Technique (SMOTE) [14] technique in used in their work to handle class imbalance. The output mRS is converted into binary class, i.e., mRS (0–5) as alive and mRS (6) as dead. Though the method achieved c-statistics = 0.865, predicting the outcome only as alive or dead, could mislead the treatment decision as intervening might cause serious disabilities. Other research does not use this type of dichotomization. Mostly, mRS ≤ 2 is considered as one class and mRS > 2 as another class. Bentley et al. [15] also employed SVM with down-sampling for imbalance on a private dataset. They trained the model on two-class data (intracranial hemorrhage or not) with 116 patients. Data include computerized tomography (CT) scans, demographic and clinical data. Area under the ROC curve (AUC) = 0.74 is the reported result in this research. Another study of 1383 patients from MR CLEAN dataset [16] with only clinical and demographic data was conducted by Van et al. [17]. They binarized the models’ output into functional dependence and independence. They used SVM, random forest classifier, artificial neural network (ANN) and an ensemble of all these models for prediction. Using only variables from data before treatment, maximum achieved AUC = 0.79. CT scans (cerebral blood volume (CBV), mean transit time (MTT)) of 512 patients were used by Xie et al. [18] to predict functional dependence and independence. All voxels participated in prediction without any feature extraction. To overcome imbalanced data, stratified sampling was used. Gradient boosting machine (GBM) and extreme gradient boosting (XGB) achieved AUC = 0.65 and AUC = 0.67, respectively. Tang et al. [19] suggested the use of radiomics features based on penumbra quantification from apparent diffusion coefficient (ADC), cerebral blood flow (CBF) for predicting clinical outcome. Seven-day mRS was dichotomized into functional clinical outcome and non-functional clinical outcome. The logistic regression model was trained on 168 patients using seven-day mRS as the availability of 90-day mRS was limited. Testing the model on 90-day mRS achieved AUC = 0.77. Lin et al. [20] trained SVM, random forest, ANN and an ensemble of these models using clinical variables at initial, as well as follow-up stages using dichotomized mRS ≤ 2 and mRS > 2. The dataset in this study was big with 35,798 samples but had high imbalance, which was dealt with by down-sampling. Without using any follow-up data, the maximum achieved AUC = 0.92. Wang et al. [21] employed logistic regression, ANN, SVM, random forest and AdaBoost for the prediction of symptomatic intracerebral hemorrhage (sICH) on 2237 samples. Oversampling and cost-sensitive adaptation was used for imbalanced distribution of sICH to no-sICH and achieved AUC = 0.82. Despite the large dataset, they suggested the need for more data to optimize the model’s performance. Bacchi et al. [22] showed the trained convolutional neural network (CNN) in combination with ANN on 204 samples for the prediction of dichotomized three-month mRS ≤ 1 and mRS ≤ 2. CT scans and clinical data served as inputs to CNN and ANN, respectively. The reported accuracy = 0.74 and F1 score = 0.69. This study states its limitation in terms of fewer data. Wang et al. [23] developed a Lasso logistic regression model for the prediction of hemorrhagic transformation (HT) at 30 days that may contribute to patient selection for therapy. Clinical data in US Electronic Health Record dataset comprising of large population of 621,178 patients was used in this study, out of which 5642 had hemorrhagic transformation (HT) showing the imbalance in the dataset. They also tested the model on various large repositories achieving the mean AUC = 0.71. Heo et al. [24] developed a deep neural network, random forest and logistic regression on 2604 patients’ clinical data. mRS at 90 days was dichotomized into favorable (mRS ≤ 2) and non-favorable outcome (mRS > 2). The patients receiving thrombolytic treatment were not included as they could hinder the process of patient selection for therapy. AUC = 0.88 was the achieved result for deep neural network. Nishi et al. [25] showed the performance of convolutional neural network using diffusion weighted imaging data of 250 patients as input for training. The mRS was dichotomized to good (mRS ≤ 2) and poor clinical outcome (mRS > 2) for model training. Data augmentation was done to counter the small dataset, but imbalance was not addressed. AUC = 0.81 ± 0.03 was reported in a five-fold cross validation result. Ling-Chien Hung et al. compared several machine learning models on demographic and clinical data to predict the risk of readmission. They mainly focused on experimenting with different techniques for handling the imbalance in data. Their study did not include any treatment-related variables of the acute stage, which may play a vital role in assessing the risk of readmission or mortality. In addition, the sampling of data from the population did not include patients treated at the acute stage [26]. Yee-Haur Mah et al. trained SVM on CT scans and clinical data to predict pre-admission and discharge mRS. The mRS values were dichotomized in low and high mRS. The SVM model achieved the AUC = 0.77 and 0.76 for both types of predictions showing the applicability of machine learning models to the problem [27]. Wenjuan Wang et al. did a review to identify and critically appraise the reporting and developing of Machine Learning (ML) models for predicting outcomes after stroke. They emphasized the need for describing the ML models sufficiently to reproduce them, so that models might be considered for practice [28].

### 2.2. Non-Dichotomized Output

Asadi et al. [29] worked on the private dataset of 107 patients without including any imaging data. SVM, linear regressor and ANN were built for predicting a clinical outcome. The results were reported on both dichotomized and non-dichotomized outputs. This work did not address the problem of imbalance despite the imbalanced dataset. Forkert et al. [30] used the SVM model with a private dataset of 68 patients. The data had MR fluid-attenuated inversion recovery (FLAIR) scans on 30 days from stroke onset and clinical data from initial stage. They incorporated lesion size and location into account using brain atlas. On scaled mRS from 0–5, the model displayed accuracy of 0.56. They used an equal number of samples for each class during model training to deal with the imbalance. This paper focuses on finding the relations in size and location of a 30-day lesion with mRS, rather than providing decision support for intervention.

Basit et al. (2016) [31] extracted handcrafted image-based features from all images per case in ISLES 2016 dataset. Random forest regressor was trained with majority class voxels, down-sampled to overcome the imbalance. Their method achieved the mean absolute error (MAE) of 1.2 ± 0.87 with the last position on ISLES 2016 challenge test set [32]. They only used imaging data before treatment, excluding clinical data.

Choi et al. (2016) [33], built three different models to predict mRS with the same dataset. This was the only work employing deep learning on this type of dataset and was ranked second in ISLES 2016 challenge. Their developed models were deep convolutional neural network, logistic regression and an ensemble of both techniques, achieving MAE of 1.37 ± 1.00, 1.26 ± 0.81 and 1.10 ± 0.70, respectively. In the ensemble technique, image-based features from a 3D CNN were combined with a logistic regression model. For training 3D CNN, the problem was modelled as a patch-wise classification problem. Three dimensional patches from training data were used for training and three-month follow-up binary segmented lesion served as ground truth available in the dataset. This pretrained 3D CNN was then used as a feature extractor, followed by a shallow fully connected network (FCN) with the last layer having five units representing mRS. In this way, the lack of data is addressed by (1) using patch-based training, such that the network is trained on many patches, and (2) using pretrained weights fixed for 3D CNN, and only FCN is trained from scratch. The number of patches near the lesions were kept the same from each mRS class to counter the imbalance. In comparison, we deal with the lack of data and imbalance by few-shot learning and two-stage balancing, respectively. They resized the images to 256 × 256 × 32, whereas in PMFE-SN images are resized to 150 × 150 × 21.

Maier et al. 2016 [34] extracted hand crafted features from multi-parametric MRI images from ISLES 2016 dataset with no preprocessing. All these features are described in [35]. The main idea of their work was to identify core, penumbra and normal brain region to estimate mRS. Using random forest classifier, lesion core was identified on ADC following a binary dilation to estimate penumbra. The rest of the ADC scan was considered normal brain. After identifying these three regions on ADC, further features were extracted from each of these regions from ADC and random regressor forest was trained for prediction. Stratified samples of voxels were used to overcome the imbalance. They did not make use of any clinical data. With the top result on ISLES challenge 2016 [29] test set, the method scored MAE = 1.05 ± 0.62.

Kabir et al. [36] used M5 model trees with boost strapping aggregating on clinical data of 437 patients. Clinical data included mRS at admission and at the time of discharge along with other data to predict full scale mRS at 90 days (0–6). The regression model achieved R = 0.822, MAE = 0.537 and RMSE = 0.832 and classification model achieved the accuracy = 59.7. Imbalance was not handled in this work despite the highly imbalanced dataset. This study can help in efficient resource allocation to manage stroke patients in hospitals rather than selection of patients for treatment. Zeynel A. Samak et al. presented an approach to predict functional outcomes using multimodal CT and clinical data. They built a deep learning model and incorporated an attention mechanism to extract features both spatially and channel-wise. Focal loss was used to deal with class imbalance. The results were reported on both dichotomized and non-dichotomized mRS with accuracy used for full scale mRS. Reporting accuracy as an evaluation metric for non-dichotomized output in the presence of class imbalance may mislead in measuring model performance [37].

From the literature review, few observations hold. First, dichotomizing the output may give the impression of better performances of the models rather than capturing the correlation of input data to each mRS score and the process of dichotomizing the mRS is not consistent throughout the studies. Secondly, there is a lot of heterogeneity in datasets, i.e., different datasets exist with different imaging modalities, demographic or clinical data. Lastly, most papers are highlighting the imbalance in small datasets, regardless of the heterogeneity in datasets.

## 3. Methodology

This research focuses on the problem of scarcity of multi-parametric MRI data and class imbalance for predicting treatment outcomes in acute ischemic stroke patients. The developed method is based on a novel strategy of treating this problem as a few-shot imbalanced classification problem due to (1) very few samples per class, (2) discrete values (mRS) for each label and (3) high imbalance in class labels. Few studies in medical imaging have exploited the dimension of few-shot learning. In this work, we draw inspiration from earlier work in few-shot learning, such as siamese network [38], also used for content-based image retrieval [39]. Siamese networks are a special type of neural network architecture. Instead of a model learning to classify its inputs, the siamese neural network learns to differentiate between two inputs by learning similarity between them [39]. This type of network basically consists of two identical neural networks, each taking one of the two inputs. The last layer of the network calculates the similarity or distance between the features extracted for two inputs. The basic siamese architecture for one shot learning [38] consists of the deep learning feature embedding for single image per sample in data. We have developed a novel parallel multi-parametric deep learning feature embedding for useful feature extraction in multi-parametric images per sample. Normalized cosine similarity is used in our work as distance metric contrasting to L1 distance used in [34]. Moreover, we have initialized the parallel convolutional layers of the developed feature embedding with pretrained ImageNet weights [40] for better learning during model training instead of random weight initialization. For handling imbalance, we developed a two-stage balancing strategy. First, stage balancing is performed at patient level to make the number of samples equal for each class. This helps to reduce the model bias towards predicting majority class, i.e., mRS. Second, stage balancing is performed at the pair level. Number of pairs from similar and dissimilar classes are made equal along with keeping the number of pairs from dissimilar class labels same. In this way, the model learns the similarity and dissimilarity in an equal manner during optimization. Moreover, pairs from dissimilar class labels are equally emphasized during training. Both these strategies are explained in detail in Section 3.2.2 and Section 3.2.3, respectively. To the best our knowledge, this approach has not been applied yet for this problem. The developed model can be helpful in other domains, as well where nature of data is multi-parametric, having an imbalance with very few samples available.

### 3.1. Data

The data consist of 43 cases from two medical centers provided in ISLES challenge 2017 (training set) [12]. Each case has an apparent diffusion coefficient (ADC) map deduced from diffusion weighted imaging (DWI), showing the infarct core. The salvageable brain tissue is represented by different perfusion maps derived from perfusion weighted imaging (PWI). These maps include mean transit time (MTT), time-to-peak (TTP), time-to-maximum (Tmax), cerebral blood volume (CBV) and cerebral blood flow (CBF). All these images are 3D volumes with different resolutions per case. Co-registration and skull stripping are already performed on all images. Clinical data are also available denoting the time since stroke (TSS), time to treat (TTT) and TICI. TSS is the time passed since stroke onset till image acquisition. TTT is the time that will still pass to treatment/intervention. TICI has been explained in Section 1 of this paper. The ground truth in this dataset is clinical outcome at 3-month follow up represented by mRS ranging from 0 to 4. As accurate predictions of treatment outcome from multi-parametric MRI data before treatment can provide invaluable evidence to support the treatment decision, only MRI scans before treatment are used in this study. Although PWI scans and TSS also belong to the data captured before treatment, neither were used in this work.

**Figure 2:**
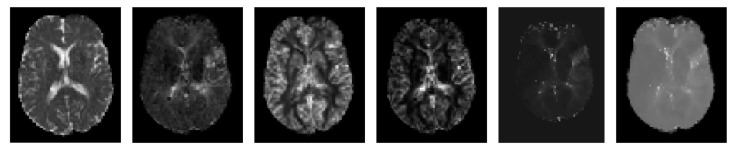
Center axial slice extracted from 3D volume resized to 150 × 150 × 21 of case no. 8. The shown maps are apparent diffusion coefficient (ADC), mean transit time (MTT), cerebral blood flow (CBF), cerebral blood volume (CBV), time-to-maximum (Tmax) and time-to-peak (TTP) (left to right).

The sample MRI scans of case no. 8 used in this work are presented in Figure 2.

### 3.2. Basic Framework

**Figure 3:**
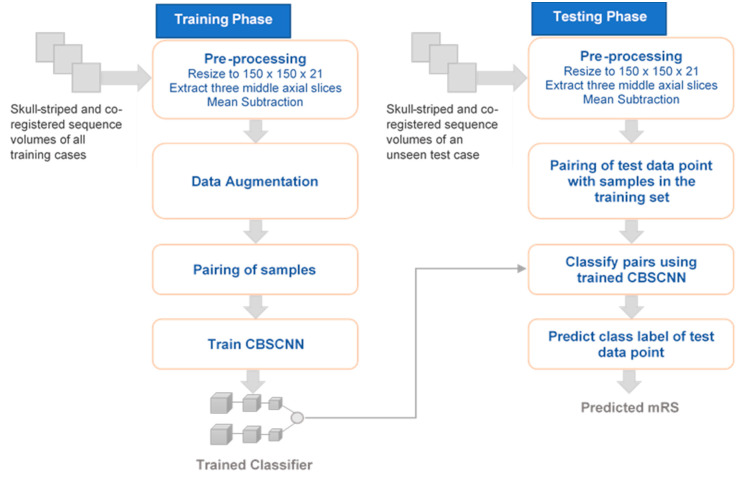
Framework for clinical outcome prediction.

The overall framework presented in this work is shown in Figure 3. During the training phase, first step is the preprocessing of training data followed by data augmentation of minority classes. Pairs are then created from augmented training set using samples belonging to similar and dissimilar classes. Next, PMFE-SN is trained using the created pairs. During testing phase, test data point is preprocessed. After preprocessing, pairs are created using test data point and each original data point in the training set, excluding the augmented samples. These pairs are then passed from the trained PMFE-SN model, which puts out a similarity score for each pair. The class of the data point in the non-augmented training set having maximum similarity with test data point is the predicted class of the test data point.

#### 3.2.1. Preprocessing

Image data are resized to 150 × 150 × 21 resolution. The images are already skull-stripped and co-registered. Bias-field correction or intensity range standardization are not required for ADC and perfusion maps [34]. Three middle axial slices from each volumetric multi-parametric MRI image per patient are extracted. In each fold of leave one cross-out testing, the mean of training data is subtracted from training, as well as from the test data.

#### 3.2.2. Data Augmentation

This is the first stage of balancing. In each fold of leave one cross-out testing, only training samples are augmented to address skewness in data. All minority classes are augmented using new samples. Geometric transformations including zoom, rotation, translation, shear, horizontal flipping and vertical flipping are applied to generate new samples. The transformations applied are the same for each multi-parametric image per sample. In this way, each class has equal number of samples before creating pairs.

#### 3.2.3. Pair Creation for Training

**Figure 4:**
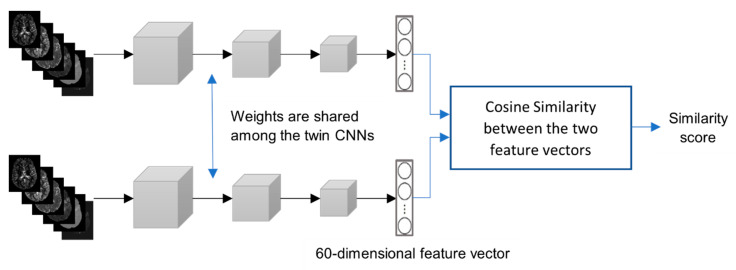
Classification model architectural diagram.

A “pair” refers to a set of any two samples in the augmented training set. It is mandatory to train PMFE-SN on pairs as it consists of two subnetworks each taking single input from a pair as shown in Figure 4. The output of PMFE-SN is normalized cosine similarity between two samples in a pair provided as input to the model. This section explains the second stage of balancing that is applied while creating pairs from same and different classes.

##### Pairs of Samples from Same Classes

Unique pairs of samples are generated from each class in the augmented training set. The formula used is $\frac{n!}{2!\left(n-2\right)!}$ where *n* is the number of samples in each class, i.e., same for all classes after augmentation. Let *S* be total number of pairs of samples from same class and is given by

(1) $$S=\;\overset{M}{\underset{m=1}{\sum }}\frac{n_{m}!}{2!\left(n_{m}-2\right)!}$$

where *M* is number of classes and *m* is the number of samples/class.

##### Pairs of Samples from Dissimilar Classes

Let *D* be the number of unique pairs of samples generated from dissimilar classes and can be calculated using (2). *D* is much larger than *S* in (1) and can lead to model bias in learning dissimilarity between samples more than learning similarity. To counter this, number of pairs from similar and dissimilar classes for model training must be same. One strategy could be random sampling of *S* number of pairs from *D* dissimilar pairs. However, it does not guarantee the selection of equal number of pairs of samples from any two dissimilar classes for training. For example, the number of sample pairs from class 0 and 1 is not necessarily equal to the number of sample pairs from class 0 and 2 via random sampling. So, the pairing strategy from dissimilar classes is as follows. Let L be the set of class labels, i.e., mRS. Make $Z=\frac{l!}{2!\left(l-2\right)!}$! unique pairs from L where *l* is the number of labels in L. Create *S*/*Z* number of pairs from samples belonging to each dissimilar class pair.

(2) D= ∑z=1ZSZ

#### 3.2.4. Classification Model Architecture

The classification model presented in this paper learns the similarity between the pairs of samples in the training set. A pair having samples from the same class is given a label 1 and a pair of samples from different class is given label 0. A siamese network consisting of two twin convolutional neural networks is trained to get features for samples in a pair belonging to the same class or belonging to different class. The word “twin” here is crucial as all the weights of both CNNs should be same as in the original work [39]. This weight sharing ensures that samples from the same class will map closer to each other and not in different parts of the embedding space as each branch of siamese has the same functionality. In addition, it makes the network symmetric, i.e., in each pair, similarity between two samples remains same irrelevant of the order of the sample in a pair [41]. Another benefit of this weight sharing is the reduction of number of parameters in the model by half. Cosine similarity is computed between the features obtained for each sample in a pair in the output layer. Cosine similarity is normalized to keep the output of the model between 0 and 1. Binary cross entropy loss function is minimized using backpropagation algorithm, stochastic gradient descent. This results in maximizing the similarity between the samples from similar class and minimizing the similarity between samples from dissimilar classes. At the test time, pairs are created of test sample with each original sample in the training set excluding the augmented training samples. The similarity is computed for each of these pairs by passing each pair from the trained PMFE-SN. The class of the sample in the training set with maximum similarity with the test data point is predicted class of test data point, i.e., predicted mRS. The classification model architecture is shown in Figure 4.

##### Parallel Multi-Parametric Feature Embedding

**Figure 5:**
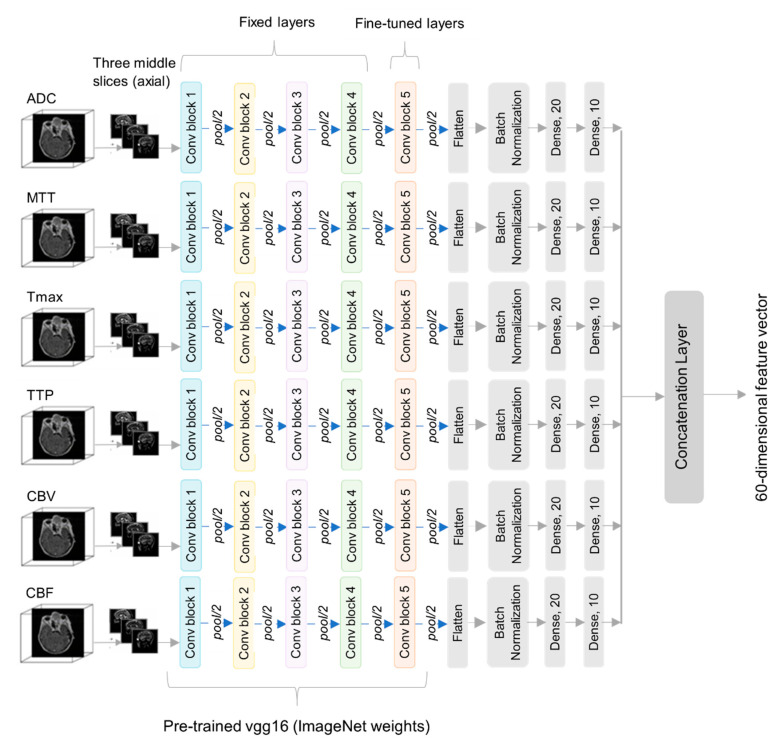
Details of single branch of parallel multi-parametric feature embedded siamese network (PMFE-SN).

Deep learning (DL) models have shown outstanding performances in the recent decade. These models can analyze complex, high dimensional and noisy data sets. DL models are deeper variants of ANNs with multiple layers. Each layer is connected to its lower and upper layers through different weights. The capability of DL models in learning hierarchical features from various types of data, e.g., numerical, image, text and audio makes them powerful. In turn, they can solve recognition, regression, semi-supervised and unsupervised problems [42,43,44]. Deep learning has proven its efficacy in medical imaging like in many other domains such as self-driving cars, natural language and image processing, predictive forecasting, eye tracking systems, object detection in space, finger print localization systems [45,46,47,48,49]. Vgg16 is one of the deep learning models [50] that is a successful feature extractor in multiple domains having lots of image data. Due to the scarcity of big data, especially in medical imaging, these deep learning models are combined with transfer learning [51]. In transfer learning, the weights from pretrained models trained on millions of image data, such as ImageNet [40], are transferred to solve other tasks having fewer data and are fine tuned. But this strategy can lead to overfit even training only the last layer of pretrained model due to very small number of samples per class. Experimenting this technique on our dataset with same data augmentation did not improve accuracy for the minority class. In PMFE-SN, a novel deep learning based multi-parametric embedding function has been developed for feature extraction. The embedding function has six parallel pretrained vgg16 models trained on ImageNet as shown in Figure 5. Each of these vgg16 models are till the last convolution block 5, excluding all the fully connected layers. Only last convolution block 5 is fine-tuned keeping all the earlier layers’ weights fixed.

This is to avoid overfitting as the amount of training data even after pairing is not very large as in the case of natural images. Each vgg16 model is then followed by a flatten layer, batch normalization layer, dense layer having 20 neurons with ReLU activation and a dense layer having 10 neurons with ReLU activation. Figure 5 provides the details of CNN used to get feature embedding from each input MRI modality per sample. Total number of parameters in PMFE-SN is 7,259,878 × 6 where 6 is the number of multi-parametric MRI per sample. The batch normalization layer is important as we empirically found that network does not learn without it. Each vgg16 is taking resized three middle axial slices from MRI volume modalities i.e., ADC, MTT, CBV, CBF, Tmax, TTP per case. The output from all vgg16 models is concatenated to make a 60-dimensional feature vector. Table 1 demonstrates the CNN for feature embeddings in detail.

##### Distance Metric and Loss Function

Normalized cosine similarity is used as the distance metric between 60-dimensional feature vector of both samples in a pair. After computing similarity, the network outputs a similarity score between 0 and 1. Binary cross entropy loss is then employed such that the similarity between the similar samples in a pair is maximized and similarity between the dissimilar samples is minimized using stochastic gradient descent. The pseudocode of PMFE-SN is shown in Algorithm 1.
**Algorithm 1.** Algorithm of PMFE-SN in leave one cross-out foldN is the number of samples in the training set X.*M* is the number of classes in the training set. $n_{m}$ is the number of samples belonging to class m.L={0,…, M−1}, the set of class labels.**Training Phase****Input:** Training set $=\left\{\left(x_{1},y_{1}\right),\ldots ,\;\left(x_{N},y_{N}\right)\right\}$, where each $y_{i}$ ∈ *L***Method:**1: Identify *I*, representing the largest number of samples amongst all classes.2: Augment each class *m* with *I*−*n_m_* number of new samples in the training set.3: Create unique pairs of samples from class *m* where m ∈{1,…,M} and put in set SP.4: Assign label 1 to each pair in set *SP*.5: Create unique number of pairs of dissimilar class labels from set *L* and put in set *LP*.6: Create $\frac{\left|SP\right|}{\left|LP\right|}$ unique pairs of samples for each dissimilar class label pairs in *LP* and put in set *DP*. 7: Assign label 0 to each pair in set *DP*.8: Let Train=SP U DP such that $Train=\left\{\left(p_{1},b_{1}\right),\ldots ,\left(p_{\left|SP\right|+\left|DP\right|},b_{\left|SP\right|+\left|DP\right|}\right)\right\}$, where *p_i_* is the *i*^th^ pair of training and *b_i_* ∈ {0,1} 9: Split Train in ratio 7:3 for training and validation of PMFE-SN. 10: Train PMFE-SN.**Testing Phase****Input:** Initial Training set $=\left\{\left(x_{1},y_{1}\right),\ldots ,\;\left(x_{N},y_{N}\right)\right\}$, where each $y_{i}$ ∈ *L*   Test Sample: $\hat{x}$
**Method:**1: Make pairs of testing sample $\hat{x}$ with every original sample in *X*.2: Pass all pairs from trained PMFE-SN to get similarity of each pair.3: Class of sample in *X* with highest similarity with $\hat{x}$ is the predicted class of $\hat{x}$.4: If there is more than 1 sample in *X* with highest similarity with $\hat{x}$, choose randomly amongst such samples and assign the class of randomly chosen sample to $\hat{x}$.

### 3.3. Evaluation Metrics

It is evident from the literature review that none of the methods for predicting non-dichotomized output have discussed the performance measures in detail for this problem. The state-of-the-art methods using imaging data are evaluated using mean absolute error (MAE) in ISLES 2016 challenge results [32]. For high class imbalanced datasets, MAE can be misleading and computing a macro averaged MAE (*MAE^M^*) across all classes is more robust [52,53]. In addition, like MAE, it also accesses the amount of deviation of true class from predicted class. This deviation is crucial to the problem at hand, as high deviated output from true class might lead to wrong decision of treatment. *MAE^M^* = $\frac{1}{M}\;\overset{M}{\underset{m=1}{\sum }}\frac{1}{n_{m}}\overset{n_{m}}{\underset{i=1}{\sum }}\left|y_{i}-{\hat{y}}_{i}\right|\;I_{\left\{y_{i}\;\in \;class\;m\right\}}$ where *M* is the number of classes, $n_{m}$ is the number of samples in $m^{th}$ class, *I*{} is the indicator function with value 1 if $y_{i}$ ∈ *class* m and 0 otherwise and *MAE^M^* ∈ [0,*M* −1]. For a fair comparison, methods are also evaluated using classification metrics for imbalanced data. These metrics include macro averaged F1 ($F1_{macro}$), macro averaged precision ($P_{macro}$), macro averaged recall ($R_{macro}$), sometimes referred to as balanced accuracy, and Matthews correlation coefficient (*MCC*). The idea of macro-averaging in $F1_{macro}$, $P_{macro}$ and $R_{macro}$ is to calculate the measure for each class separately and then take the average of these measures. In this way, all classes are weighted equally, regardless of their sample size. This averaging is important to assess a method, whether it can classify rare, as well as common classes [53]. Computing $R_{macro}$ for this problem refers to measuring the ability of a method to avoid deviation from true mRS, but like *MAE^M^*, it cannot calculate the amount of deviation from true mRS. Computing $P_{macro}$ for this problem is important to see if the classifier has a tilt towards predicting an mRS due to a greater number of samples. $F1_{macro}=2\;\frac{P_{macro}R_{macro}}{P_{macro}+\;R_{macro}}$, where $P_{macro}=\;\frac{\sum_{m=1}^{M}P_{m}}{\left|M\right|}$ and $P_{m}$ is the precision of class *m*, $R_{macro}=\;\frac{\sum_{m=1}^{M}R_{m}}{\left|M\right|}$ and $R_{m}$ is the recall of class m. MCC for multi-class is described in detail by Gorodkin [54] and is a suggested metric to measure the performance of classifiers for imbalanced data and has been extensively used in bioinformatics [55]. Area under the ROC curve (AUC) is also reported for each class.

## 4. Experimental Setup

All experiments are performed using Intel (R) Zeon (R) Silver 4210 CPU @ 2.20 GHz, 2.19 GHz (two processors), NVIDIA Tesla P40 Graphics Card, 128 GB RAM, Gryphon Z87 Motherboard, Windows Server 2016 standard operating system, Keras and TensorFlow-gpu. Learning rate of 0.0001 is used with a momentum = 0.9 and batch size = 32. In every fold, the model is trained for 20 epochs with an early stop when the decrease in training loss is equal to or less than 0.0001 using patience = 1. Stochastic gradient descent backpropagation optimizer is used to train the network. Data are shuffled before each mini batch training iteration. All the weights, except the convolutional layers, are initialized using random normal distribution with the mean = 0 and standard deviation = 0.01 [34].

## 5. Results and Discussion

**Figure 6:**
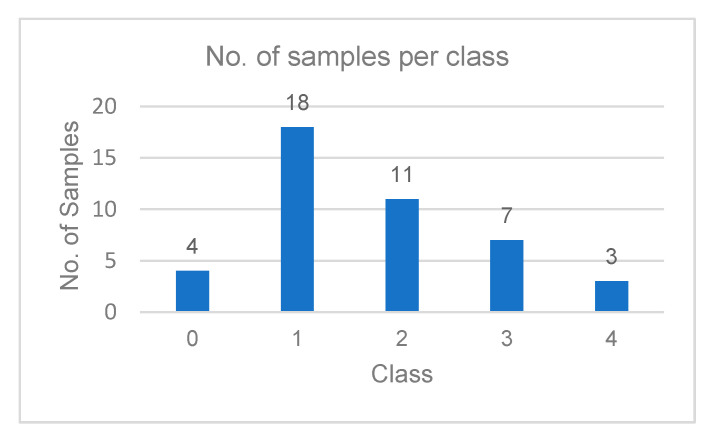
Bar chart showing number of samples per class.

The proposed PMFE-SN in this work is compared with state-of-the art methods on ISLES 2017 challenge dataset with 43 samples [12]. Considering the small dataset, the results are reported on leave one cross-out testing. The dataset is not only highly skewed but the number of samples for class 4 is smaller, as shown in Figure 6.

**Figure 7:**
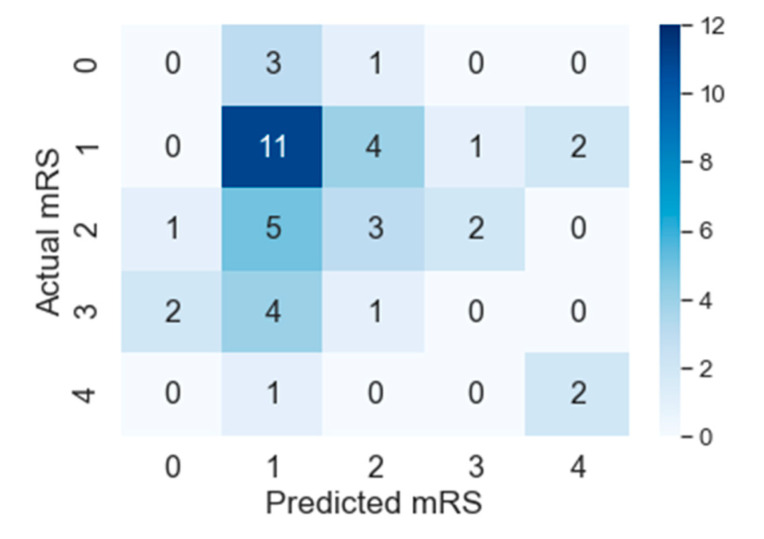
Confusion matrix of PMFE-SN on leave one cross out testing.

The results reported in this section show that PMFE-SN is outperforming the state-of-the-art in all metrics suitable to this problem. The top method amongst the state-of-the-art methods in ISLES 2016 challenge is using random forest regressor on hand crafted features and its output values are continuous. For computing *MAE^M^*, these continuous output values are not rounded off to discrete values. PMFE-SN, on the other hand, has the output in the form of discrete class label as it models the problem as a classification problem. *MAE^M^*, for PMFE-SN is hence computed using the confusion matrix in Figure 7. The results in Table 2 show that PMFE-SN is performing better than state-of-the art in terms of *MAE^M^*.

**Figure 8:**
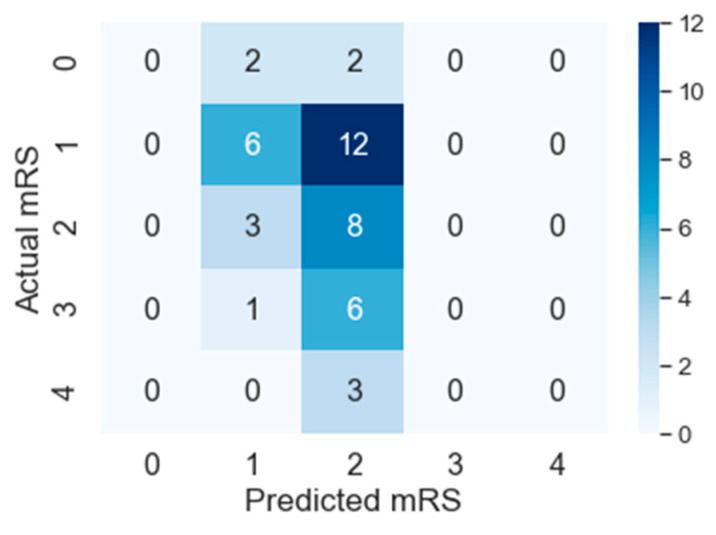
Confusion Matrix of state-of-the-art on leave one cross out testing.

$F1_{macro}$, $P_{macro}$ and $R_{macro}$ and *MCC* are computed using confusion matrices for both methods provided in Figure 7 and Figure 8. The continuous output from state-of-the art is rounded off to compute the confusion matrix (see Figure 8). It is important to note that all confusion matrices are constructed using leave one cross out testing. The results on all the classification metrics for both methods are provided in Table 3. Clearly, PMFE-SN has improved performance on all the metrics.

Table 4 demonstrates per class precision, recall, accuracy, and AUC for both methods. All the values in Table 4 are also computed using the confusion matrices in Figure 7 and Figure 8. PMFE-SN has mostly better precision and recall per class. Moreover, it is seen from confusion matrices that PMFE-SN can predict the class (class 4) with least number of samples (see Figure 7) but state-of-the-art cannot predict any samples of this class (see Figure 8).

Class accuracy reported in Table 4 shows the accuracy improved by PMFE-SN in predicting class 4 (with minimum number of samples), as well as class 1 (with maximum number of samples). This result depicts that the model is not biased towards predicting the majority class. It has improved the accuracy of the majority, as well as minority class. Class 4 and class 1 are predicted with 67% and 61% accuracy, respectively, in comparison to state-of-the-art with 33% accuracy for class 1 and no correct prediction for class 4. Hence, PMFE-SN is learning with very few and imbalanced data. Moreover, since most of the cases are from class 1 and class 2, and state-of-the-art method is predicting every sample as belonging to class 1 or class 2 (see Figure 8). This shows that state-of-the-art method is biased towards predicting majority class. PMFE-SN on the other hand can predict the outcome in the range 0–4 (see Figure 7).

**Figure 9:**
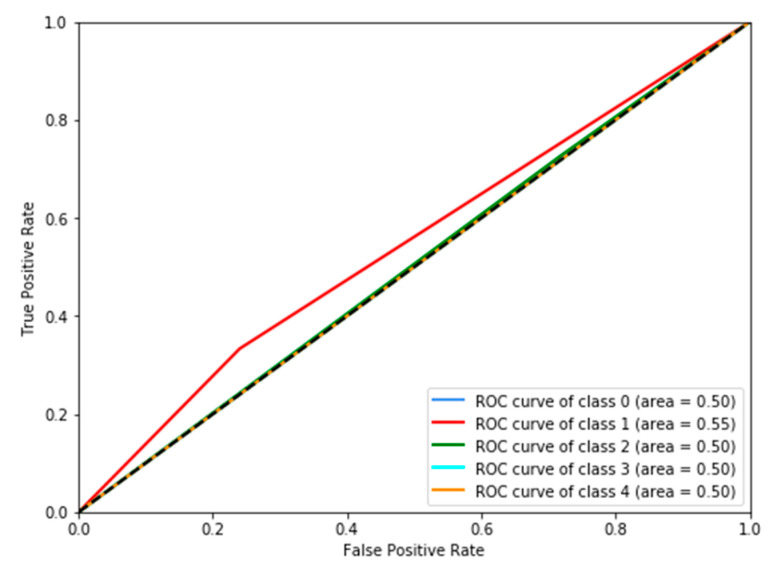
Per-class AUC of state-of-the-art.

**Figure 10:**
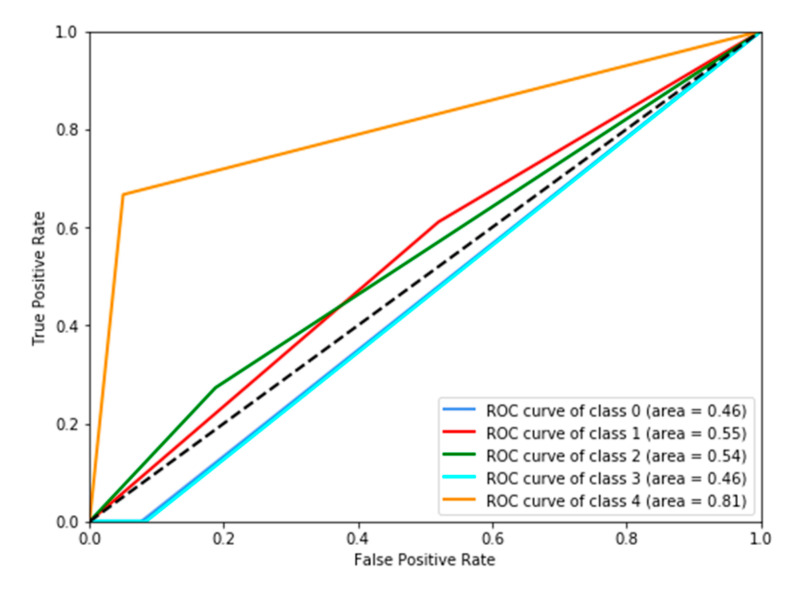
Per-class AUC of PMFE-SN.

Figure 9 and Figure 10 show AUC per class for both models. The predictive power of PMFE-SN as compared to state-of-the-art is better. It can classify and separate the minority outcome (class 4).

### Effect of Augmentation

The utility of augmenting training data in PMFE-SN is evident from the results in this section. With augmentation, PMFE-SN is performing much better in all metrics defined for this problem as compared to no augmentation (see Table 5). It is empirically found that using augmentation there is always a single sample in training data with maximum similarity with the test data point at test time. Without augmentation, there could be more than one sample with maximum similarity with the test data point. So, out of all these samples select a sample randomly and assign its class to test data point (see step 4 from testing phase of Algorithm 1 in Section 3). This means that PMFE-SN is learning similarity better in case of augmentation as compared to without augmentation.

**Figure 11:**
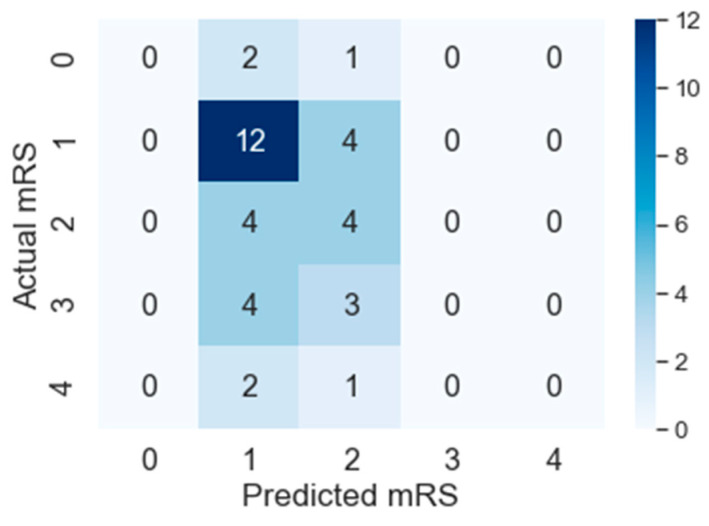
Confusion matrix of PMFE-SN without augmentation.

**Figure 12:**
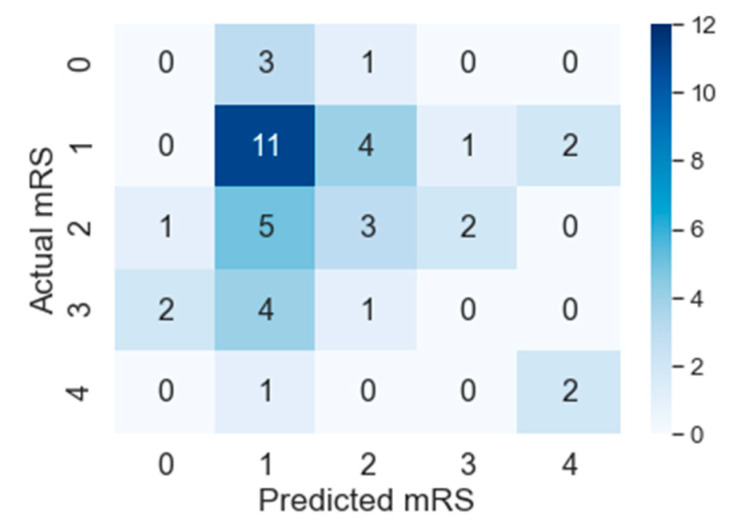
Confusion matrix of PMFE-SN with augmentation.

The confusion matrix in Figure 11 depict that without augmentation PMFE-SN mostly predicts the most occurring classes in the dataset and is contributing to model bias to predict majority class. But with augmentation PMFE-SN has predictions with all classes (see Figure 12).

Without augmentation, the accuracy is 0 for minority class, whereas with augmentation this metric is satisfactory for minority, as well as majority classes (see Table 6).

The results for state-of-the-art methods are computed and compared by ISLES 2016 challenge organizers, not by the authors, so in this paper PMFE-SN is only compared to the top result from ISLES challenge 2016. Table 7 presents methodologies and corresponding scores using cases from ISLES 2016, highlighting the top result.

**Figure 13:**
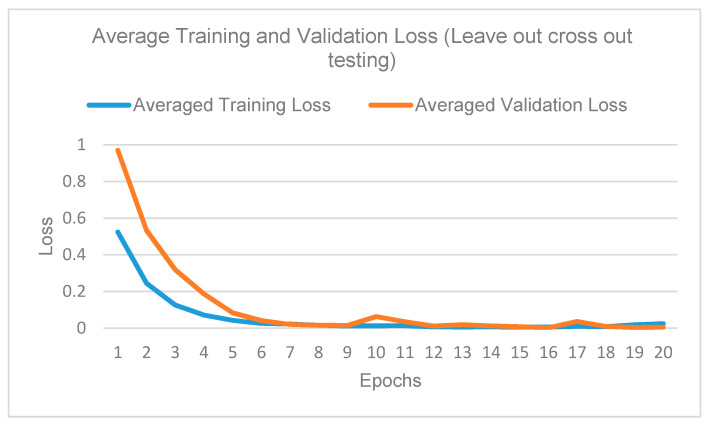
Training and validation curve for PMFE-SN on leave one cross out testing.

Figure 13 shows the training and validation curves for PMFE-SN. Due to training of only last convolution block of vgg16 in each sub network of PMFE-SN instead of training full vgg16, the model is not overfitting. But at the same time, class 0 and 3 are not predicted by PMFE-SN like state-of-the-art method.

## 6. Conclusions and Future Work

In this paper, PMFE-SN is proposed for prediction of three-month treatment outcome of patients in acute ischemic stroke. The treatment window is of few hours and involves high risk. Accurate prediction of treatment outcome with merely acute multi-parametric MRI can guide clinicians in making decisions for intervention. The multi-parametric MRI datasets for this problem are scarce and have two major issues. Firstly, these datasets are small; secondly, they have high class imbalance. PMFE-SN deals with both issues by combining parallel multi-parametric feature embedded few-shot learning with two-stage balancing strategy. The multi-parametric embedding architectural design presented in PMFE-SN is based on deep learning, but it does not suffer from overfitting even with a very small number of samples in the dataset. In addition, it is learning with even two samples in a training set. The two-stage balancing incorporated reduces model bias towards predicting majority class, as well as helps in learning from same and different class pairs in a balanced way. Evaluation metrics used in previous research may mislead in measuring the performance of various models as they are sensitive to imbalance. In addition to development of PMFE-SN, we define five evaluation metrics insensitive to imbalance for assessment of the models. Results show that PMFE-SN outperformed the state-of-the-art methods in all these metrics.

In future, more layers of vgg16 can be trained to predict class 0 and class 3 that have not been predicted correctly yet. In addition to this, training can be done using all slices instead of only three middle axial slices to exploit all the information in MRI volumetric data. Clinical data in this dataset before treatment and after treatment can be used to assess whether they play any role in improving the performance of prediction. Similarity metrics other than cosine similarity can be used for improvement in results.

## Figures and Tables

**Table 1:** Details of convolutional neural network used to get feature embedding for each magnetic resonance imaging (MRI) modality per sample.

Layer (Type)	Output Shape	No. of Parameters
input_1 (InputLayer)	(None, 150, 150, 3)	0
block1_conv1 (Conv2D)	(None, 150, 150, 64)	1792
block1_conv2 (Conv2D)	(None, 150, 150, 64)	36,928
block1_pool (MaxPooling2D)	(None, 75, 75, 64)	0
dense_1 (Dense)	(None, 75, 75, 128)	73,856
block2_conv2 (Conv2D)	(None, 75, 75, 128)	147,584
block2_pool (MaxPooling2D)	(None, 37, 37, 128)	0
block3_conv1 (Conv2D)	(None, 37, 37, 256)	295,168
block3_conv2 (Conv2D)	(None, 37, 37, 256)	590,080
block3_conv3 (Conv2D)	(None, 37, 37, 256)	590,080
block3_pool (MaxPooling2D)	(None, 18, 18, 256)	0
block4_conv1 (Conv2D)	(None, 18, 18, 512)	1,180,160
block4_conv2 (Conv2D)	(None, 18, 18, 512)	2,359,808
block4_conv3 (Conv2D)	(None, 18, 18, 512)	2,359,808
block4_pool (MaxPooling2D)	(None, 9, 9, 512)	0
block5_conv1 (Conv2D)	(None, 9, 9, 512)	2,359,808
block5_conv2 (Conv2D)	(None, 9, 9, 512)	2,359,808
block5_conv3 (Conv2D)	(None, 9, 9, 512)	2,359,808
block5_pool (MaxPooling2D)	(None, 4, 4, 512)	0
flatten_1 (Flatten)	(None, 8192)	0
batch_normalization_1 (BatchNormalization)	(None, 8192)	32,768
dense_1 (Dense)	(None, 20)	163,860
dense_2 (Dense)	(None, 10)	210
Total params: 14,911,526 Trainable params: 7,259,878 Non-trainable params: 7,651,648		

**Table 2:** Comparison of *MAE^M^* of state-of-the-art and PMFE-SN on leave one cross out testing.

Method	Cases from ISLES 2017	*MAE^M^*
Random Forest for Stroke Lesion and Clinical Outcome prediction [22]	43/43	1.24
PMFE-SN	43/43	**1.18**

**Table 3:** Comparison of $P_{macro}$, $R_{macro}$, $F1_{macro}$ and Matthews correlation coefficient (MCC) of state-of-the-art and PMFE-SN on leave one cross out testing.

Method	Cases from ISLES 2017	$P_{macro}$	$R_{macro}$	$F1_{macro}$	MCC
Random Forest for Stroke Lesion and Clinical Outcome prediction [22]	43/43	0.152	0.21	0.18	0.04
PMFE-SN	43/43	**0.258**	**0.31**	**0.28**	**0.09**

**Table 4:** Comparison of per class precision, recall, accuracy, and area under the curve (AUC) of state-of-the-art and PMFE-SN on leave one cross-out testing.

		Class				
Method		0	1	2	3	4
State-of-the-art	Precision	0	**0.50**	0.26	0	0
	Recall	0	0.33	**0.73**	0	0
	AUC	**0.50**	0.55	0.50	**0.50**	0.50
	Accuracy	0	0.33	**0.73**	0	0
PMFE-SN	Precision	0	0.46	**0.33**	0	**0.50**
	Recall	0	**0.61**	0.27	0	**0.67**
	AUC	0.46	0.55	**0.54**	0.46	**0.81**
	Accuracy	0	**0.61**	0.27	0	**0.67**

**Table 5:** Comparison of PMFE-SN with and without data augmentation using *MAE^M^*, $P_{macro}$, $R_{macro}$, $F1_{macro}$ and MCC on leave one cross out testing.

PMFE-SN	*MAE^M^*	$P_{macro}$	$R_{macro}$	$F1_{macro}$	MCC
With data augmentation	**1.18**	**0.258**	**0.31**	**0.28**	**0.09**
Without data augmentation	1.45	0.162	0.21	0.18	0.07

**Table 6:** Comparison of per-class accuracy of PMFE-SN with and without augmentation on leave one cross-out testing.

	Accuracy Per Class (PMFE-SN)				
	0	1	2	3	4
Without Augmentation	0	0.33	0.73	0	0
With augmentation	0	**0.61**	0.27	0	**0.67**

**Table 7:** ISLES 2016 challenge result showing the state-of-the-art method as top result [12].

Method	Cases from ISLES 2016	MAE
1. Prediction of ischemic stroke lesion and clinical outcome in multi-modal MRI images using random forests [31]	19/19 (test set)	1.26 ± 0.87
2. Ensemble of deep convolutional neural networks for prognosis of ischemic stroke [33]	19/19 (test set)	1.10 ± 0.70
3. Predicting stroke lesion and clinical outcome with random forests [34]	19/19 (test set)	1.05 ± 0.62
